# Identification of Behaviour in Freely Moving Dogs (*Canis familiaris*) Using Inertial Sensors

**DOI:** 10.1371/journal.pone.0077814

**Published:** 2013-10-18

**Authors:** Linda Gerencsér, Gábor Vásárhelyi, Máté Nagy, Tamas Vicsek, Adam Miklósi

**Affiliations:** 1 Department of Ethology, Eötvös Loránd University, Budapest, Hungary; 2 Department of Biological Physics, Eötvös Loránd University, Budapest, Hungary; 3 Statistical and Biological Physics Research Group, Hungarian Academy of Sciences, Budapest, Hungary; 4 Department of Zoology, University of Oxford, Oxford, United Kingdom; 5 MTA-ELTE Comparative Research Group, Budapest, Hungary; Cajal Institute, Consejo Superior de Investigaciones Científicas, Spain

## Abstract

Monitoring and describing the physical movements and body postures of animals is one of the most fundamental tasks of ethology. The more precise the observations are the more sophisticated the interpretations can be about the biology of a certain individual or species. Animal-borne data loggers have recently contributed much to the collection of motion-data from individuals, however, the problem of translating these measurements to distinct behavioural categories to create an ethogram is not overcome yet. The objective of the present study was to develop a “behaviour tracker”: a system composed of a multiple sensor data-logger device (with a tri-axial accelerometer and a tri-axial gyroscope) and a supervised learning algorithm as means of automated identification of the behaviour of freely moving dogs. We collected parallel sensor measurements and video recordings of each of our subjects (Belgian Malinois, N=12; Labrador Retrievers, N=12) that were guided through a predetermined series of standard activities. Seven behavioural categories (lay, sit, stand, walk, trot, gallop, canter) were pre-defined and each video recording was tagged accordingly. Evaluation of the measurements was performed by support vector machine (SVM) classification. During the analysis we used different combinations of independent measurements for training and validation (belonging to the same or different individuals or using different training data size) to determine the robustness of the application. We reached an overall accuracy of above 90% perfect identification of all the defined seven categories of behaviour when both training and validation data belonged to the same individual, and over 80% perfect recognition rate using a generalized training data set of multiple subjects. Our results indicate that the present method provides a good model for an easily applicable, fast, automatic behaviour classification system that can be trained with arbitrary motion patterns and potentially be applied to a wide range of species and situations.

## Introduction

The outstanding developments in technology (such as miniaturization or digital information processing) over the past few decades have provided scientists with new, yet unexploited opportunities for studying undisturbed animal behaviour apart from direct human observation. The application of suitable animal-borne sensor tags, referred to as bio-logging [1-3] offers the possibility of collecting physical (e.g., position, movement patterns) [4-7] and biological (e.g., body temperature, heart rate) [8,9] data about the tagged animal and/or even its environment [10-12]. The main original aim of bio-logging was to take measurements from undisturbed free-ranging wild animals in order to find out previously unattainable details about their lives [6,10,13]. The gathered information proved to be important in conservation issues [12,14,15], and is even relevant in the case of domestic animals to help the improvement of husbandry methods [16-18].

Monitoring and describing the physical movements and body postures of animals to create species specific ethograms is the first crucial step towards understanding animal behaviour. With the help of bio-logging the whole process of studying the structure of behaviour can become automated. Thus the need for direct visual observation by skilled human investigators to generate ethograms could be decreased. This does not only help to overcome situations when the subject of interest is beyond the limits of human vision (e.g., nocturnal, free-ranging wild animals, working dogs out of sight) but also offers a more objective way of quantifying animal activity with a significantly increased amount of available data, enhancing comparison across individuals and species. Additionally, it opens up new channels of measurements, e.g., to make interpretations about the energy expenditure of living creatures [18,13,19-21]. However, this range of new opportunities implies significant methodological challenges that are yet to be overcome, such as applying the most suitable type of sensor device(s) and finding adequate ways of analysing the measured data. 

When targeting automated behaviour identification, animal-attached accelerometers have the potential for being good indicators and discriminators of a variety of activities. Accelerometers can measure both static and dynamic body accelerations (DBA) [6,22] along one [5] or more axes [6,23,24]. Such instruments have already been successfully used on a wide range of species by researchers investigating terrestrial [5,23,25,26], underwater [3,24,27] or even airborne locomotion [28-31].

Increasing the number of attached sensors enables for a more detailed monitoring of individual behaviour. One possibility is to combine an accelerometer with a gyroscope, a device measuring angular velocity. To the authors’ present knowledge there have only been a few attempts so far to use information based on data from both accelerometers and gyroscopes in order to determine animal movement characteristics [7,22,32]. However, some evidence already suggests that gyroscope measurements can aid accelerometer-based movement analysis in dynamic, high-frequency motion situations [32].

The main advantages of accelerometers and gyroscopes as sensors for motion analysis are the following: i) they are small, lightweight, energy efficient and cheap, compared to image-analysis based motion recognition systems; ii) they can provide high frequency data about the motion of the subject at up to a few kHz, compared to e.g., a GPS (Global Positioning System) with a maximum of 5-10-20 measurements per second; iii) unlike GNSS (Global Navigation Satellite System) or GSM (Global System for Mobile Communications) chips that rely on a complex global infrastructure of satellites or radio stations to measure the position or velocity of the target, inertial sensors can function without any external aid. On the other hand, gyroscope and accelerometer data will always be local, relative and short term, compared to global position/velocity data, such as from a GPS. However, taking into account all these features, we propose that a combined system of accelerometer and gyroscope allows for the robust identification of behaviour categories of freely moving animals.

The domestic dog (*Canis familiaris*) is an ideal subject for bio-logging experiments because it naturally lives together with humans. Also, increasing our knowledge about their remote activities could potentially be used for further improving their contribution to human needs in various situations (e.g., guard dogs, search and rescue dogs). Animal-attached tri-axial accelerometers have been used before to study aspects of laboratory, companion or working dogs’ behaviour. Most of the work is linked to veterinary medicine and aims at recording locomotor activity rhythms [26,33] to determine the degree of daily activity [34-36], consequential maintenance energy requirements [21] or investigating gait patterns [37] as part of kinematic motion analysis [38]. Preliminary open-field trials on a few individuals were made to gain sensor information on dog’s location, movements, orientation, or pose [7,39]. To the authors’ present knowledge there have been no systematic investigations using an accelerometer and a gyroscope as an animal-borne multiple sensor tag aiming at the detailed automated classification of the behaviour in freely moving dogs.

The present study is based on a custom-made multiple sensor data-logger device with a tri-axial accelerometer and a tri-axial gyroscope [40] originally developed for tracking homing pigeons [41]. We extended the logger’s software framework to be used specially for our behaviour tracking purposes. Our objective was to create automatic ethograms of freely moving dogs on open field, using supervised learning algorithms trained with inertial sensor data. We have collected motion data as well as video recordings of the behaviour of 24 dogs belonging to two different breeds (Belgian Malinois and Labrador Retrievers). Videos were coded by a trained human observer, and her behaviour classifications together with the sensor data were used to train the support vector machine (SVM) based supervised learning algorithm. Various validation experiments were run in order to determine the robustness of the application and the data base collected.

We hypothesized that behaviour identification would be most successful when using training data collected from the same individual. Tests for existing comparable differences across individuals and breeds (Malinois and Labrador Retrievers) were targeted as well. We found that our system of the multiple sensor data-logger and its software framework provides a good solution for creating an automatic dog ethogram and is sensitive to individual differences in motion characteristics.

## Materials and Method

### Ethics statement

Non-invasive studies on dogs are currently allowed to be done without any special permission in Hungary by the University Institutional Animal Care and Use Committee (UIACUC, Eötvös Loránd University, Hungary). The currently operating Hungarian law “1998. évi XXVIII. Törvény” – the Animal Protection Act – defines experiments on animals in the 9^th^ point of its 3^rd^ paragraph (3. §/9.). We also obtained a written statement (XIV-I-001/526/2012) from the Food chain Safety and Animal Health Directorate Government Office based on the decision of the Scientific Ethic Council of Animal Experiments. According to this statement and the corresponding definition by law, our non-invasive observational study is not considered as an animal experiment. 

Owners with their dogs from Pannon Search and Rescue Dog Team and others responding to our advertisement at the department’s homepage (http://kutyaetologia.elte.hu) volunteered to participate.

### Subjects

Our subjects were 24 healthy adult dogs from the two different dog breeds Malinois (N=12, 9 males and 3 females; age range: 1.5–10 years, mean age: 4.0 years, SD=2.8 years) and Labrador Retrievers (N=12, 6 males and 6 females, age range: 1.5–9 years, mean age: 5.3 years, SD=2.2 years). Both breeds are frequently used in search and rescue missions. Each individual was examined by a veterinarian prior to the enrolment to the study and was found to be in healthy body condition, free of any orthopaedic and neurological disorder. Additional criteria for enrolment of a dog was being trained enough to walk without leash freely on open field and perform several tasks reliably under the guidance of the handler. 

### Inertial data logger

Inertial data was gathered with a custom-designed miniature GPS/INS (Inertial Navigation System) logger [40,41] capable of recording tri-axial accelerometer (±6g, 16 bit, ±10 LSB noise) and tri-axial gyroscope (± 500 °/s, 16 bit, ±20 LSB noise) data at 100Hz, synchronized with GPS data at 10Hz. Loggers were pre-calibrated with an XSens MTi-G device [42] to compensate for linear sensitivity, axis misalignment and cross-axis error, but were not temperature compensated. For this study GPS data only served as a synchronization method for precise timing and as an independent measurement source for validation purposes, using the velocity data. The logger was later connected to a computer via USB cable and the collected data was downloaded for off-line analysis.

### Data collection (protocol)

Data was collected in Hungary between October 2011 and April 2012 at 10 different open field locations in Budapest and in its vicinity. All outdoor terrains were plain and grassy with no major surface roughness and covered approximately at least the size of a tennis court.

The data collecting procedure consisted of 4 phases; (1) Preparation, (2) Sensor synchronisation, (3) Behaviour recording and (4) Sensor synchronisation. The whole procedure was repeated from phase 2 to 4 two consecutive times with each individual on the same occasion separated by a resting phase of 10-15 minutes.

**Figure 1:**
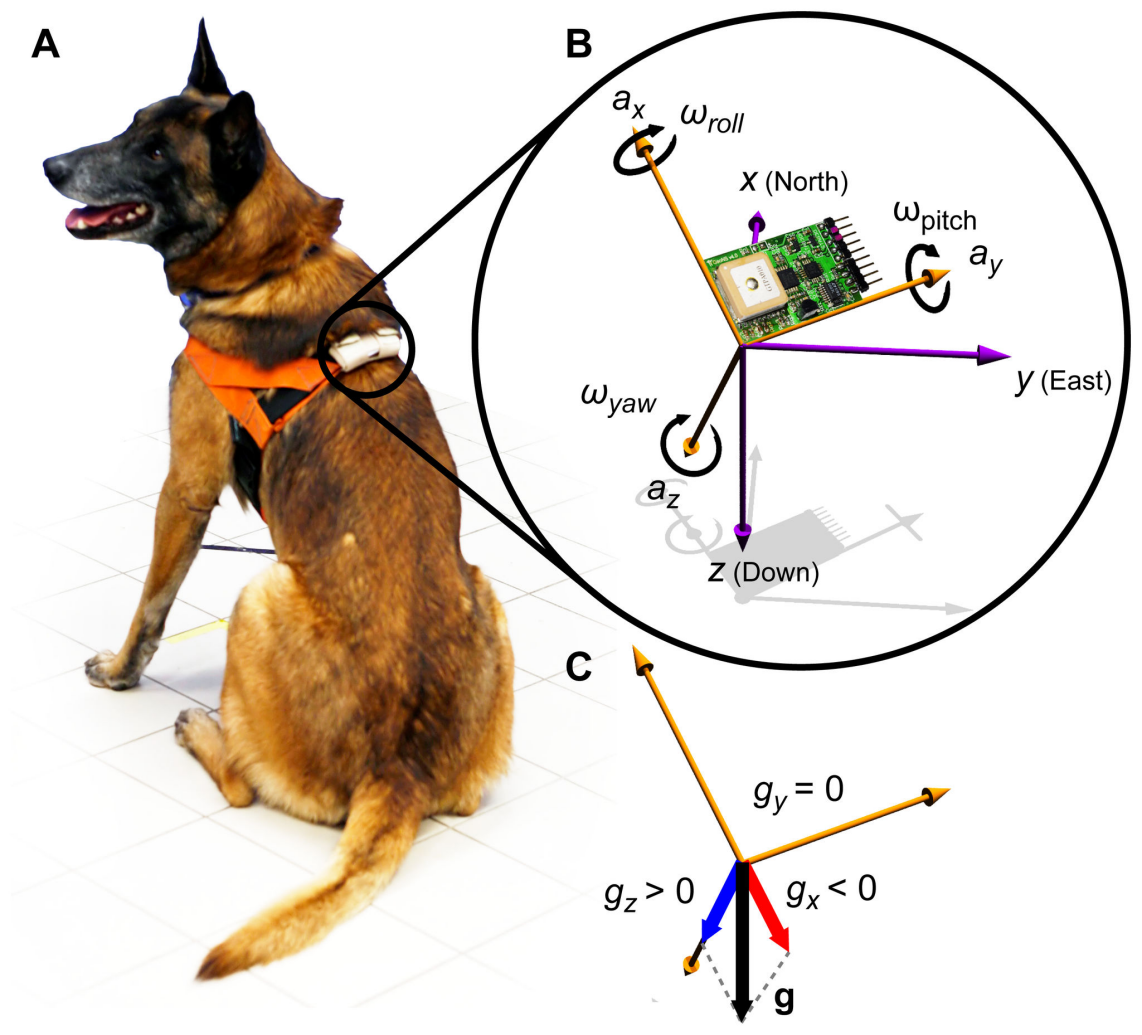
Illustration of the device and the relevant axes of the accelerometer and gyroscope. (A) Photo of a subject dog wearing the inertial data logger device fixed to an adjustable harness. The logger always stayed at a fixed anatomical position, dorsally midway between the two scapulae. (B) Photo of the actual logger device combined with the illustration of the main axes. The purple axes represent the Earth-fixed, local North-East-Down Cartesian coordinate system (NED), the orange axes belong to the body-fixed coordinate system (BFCS), which is fixed to the device and the subject. The *x* axis of the accelerometer (a_x_) points towards the head of the dog, the *y* axis (a_y_) points towards the right side, while the *z* axis (a_z_) points towards the body. The axes of the gyroscope are similar, roll, pitch and yaw represent the rotations about the BFCS axes *x*, *y*, *z*, respectively. (C) In a steady position (sitting) as shown on panel B the accelerometer measures only the gravitational acceleration (*g* with black arrow). Its components in the BFCS are shown, *g*_*x*_ (red) and *g*_*z*_ (blue), while in the current example *g*_*y*_=0.

1. Preparation. The dogs were equipped with the inertial data logger device by means of a standard adjustable harness designed and prepared specially for this purpose to fit each individual appropriately without causing any discomfort during locomotion. The data logger always stayed at a fixed anatomical position, dorsally midway between the two scapulae with its longer side perpendicular to the vertebral column (Figure 1). A firm plastic case prepared for this purpose was permanently attached to the harness where the logger could be placed, fixed and also removed from easily. From five available loggers we chose randomly for each measurement.
2. Sensor synchronisation. Once the harness with the activated logger was mounted on the dog, uninterrupted video camera recording began with a synchronisation phase. To get the precise time of the video recording another data logger was attached to a laptop to display the actual Coordinated Universal Time (UTC) measured by the logger’s GPS module. The laptop screen displaying the time in UTC was video recorded. GPS and inertial data were synchronized in the logger itself, but as an additional, independent source of synchronisation, the data logger attached to the dog’s harness was also filmed while shaken manually several times in both cranio-caudal and latero-lateral directions relative to the dog.
3. Behaviour recording. Right after the synchronisation phase, each dog was lead through a predetermined series of activities by its handler, which lasted approximately 10 minutes. Since the dogs were moving freely on open field, a continuous strict control for their behaviour was not always possible and was neither expected. The handlers were asked to instruct their dogs to perform the following tasks in a predetermined order, allowing for any desired reward for good performance in between (e.g., play, food and praise): sit, lay, stand, run, trot, walk, bark and search. The dog’s behaviour was recorded by a hand-held video camera. For detailed description and illustration of the different activities, see the Supporting Information (Table S1 and also Video S1).
4. Sensor synchronisation: at the completion of the recording of the dog’s activities, a second synchronisation phase followed, with first shaking the logger device and then recording the displayed actual time in UTC as described previously.

We performed two subsequent measurements with our subjects resulting in two independent sets of data belonging to each subject; two video recordings (Video 1 and Video 2) and the corresponding data collected by sensors on the logger (GPS, accelerometer and gyroscope). Later on subtitles were added about the behaviour (Subtitle1 and Subtitle2) to each video recording. For a short illustration of the data collecting procedure see the Supporting Information (Video S1). 

### Video recording, subtitling, behaviour categories

In addition to the inertial measurements, a hand-held video camera was used to record the scene (image size: 1280x720, frame rate: 30 frame/s). During the off-line data analysis process, video recordings were manually synchronized to the inertial data using the synchronization patterns from the measurement protocol. Commercial subtitle editor software, Subtitle Workshop Version 2.51 [43] was used to tag the video recordings with predefined motion pattern categories of the dog behaviour.

**Figure 2:**
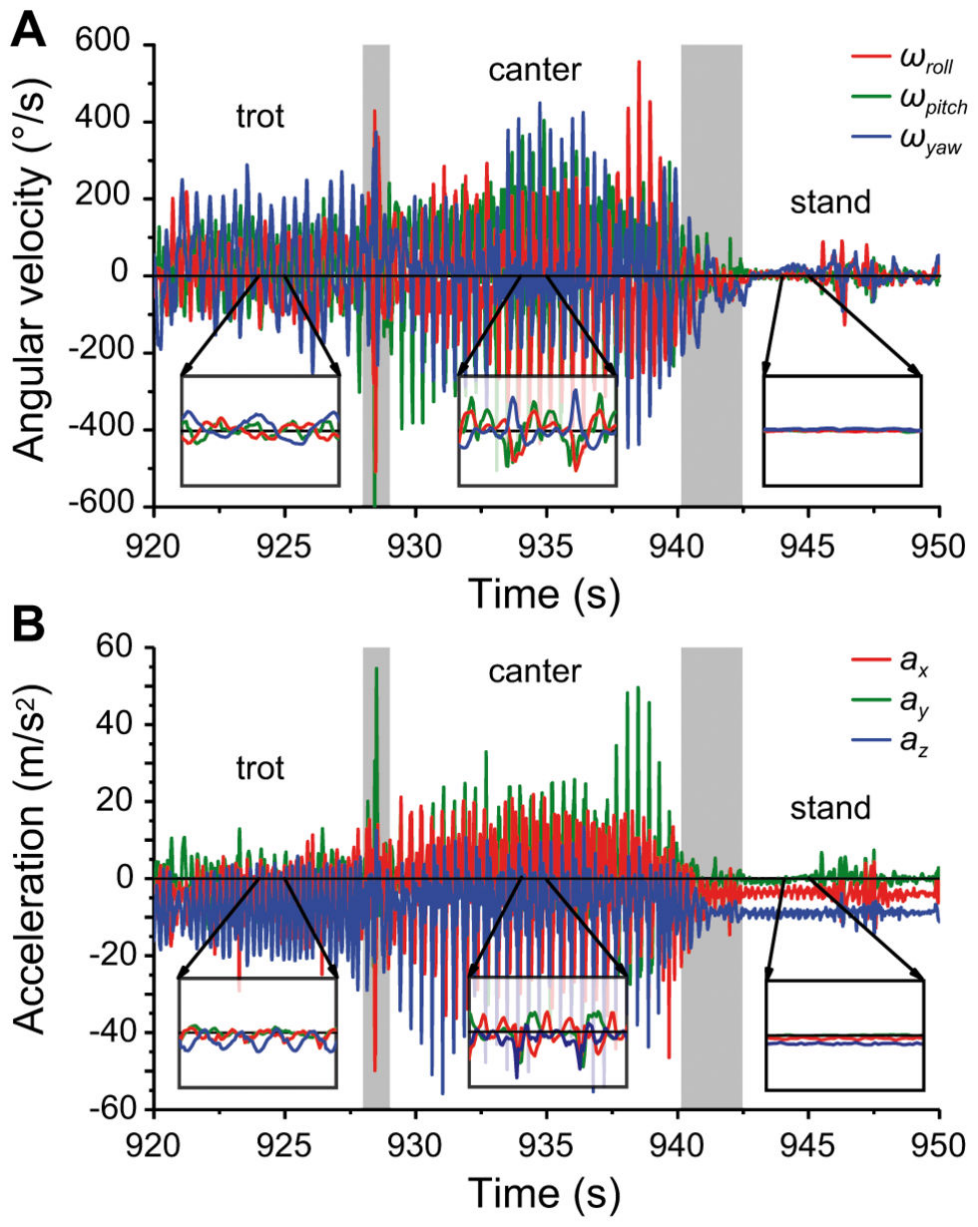
Snapshots of the raw data measured by the gyroscope (A) and the accelerometer (B). Figure shows a half-minute period of a measurement collected from a freely moving dog equipped with the data logger device. The dog performed consecutive periods of trot, canter and stand type categories of behaviour. Behaviour categories belonging to parts of the data are indicated above the curves. Insets below the curves show one second periods of the data with the same amplitude range as the main plot. Note the non-zero offset of the acceleration components due to the measured gravity during “stand”, or the high frequency of the data during active motion. Data acquisition rate is 100Hz. The colour coding of the curves refers to the different axes of the tri-axial sensors: acceleration along and angular velocity (roll, pitch and yaw) about the *x*, *y* and *z* axis shown with red, green and blue, respectively.

We defined seven basic non-overlapping behavioural categories we wished to include in our discriminative analysis, which were: stand, sit, lay, walk, trot, canter and gallop. For the purpose of later analysis we enrolled them into four *ad-hoc* activity level categories (for the definition of each category and the corresponding activity levels, see Table S1). Motion pattern tags referring to the defined categories were added to the proper time period of each individual video recording with the subtitle editor software. Only the objectively definable sections of the videos, i.e., only the clearly distinguishable dog behavioural elements were tagged, resulting in interrupted but unequivocally subtitled recording. For illustration of the labelled, raw data measured by the sensors see Figure 2. All video recordings were tagged by the same, main coder and 50% of the recordings used for the latter calculations (Video 1 and Video 2 of five Labradors and five Malinois) were additionally tagged by a secondary coder. 

### Data analysis

**Figure 3:**
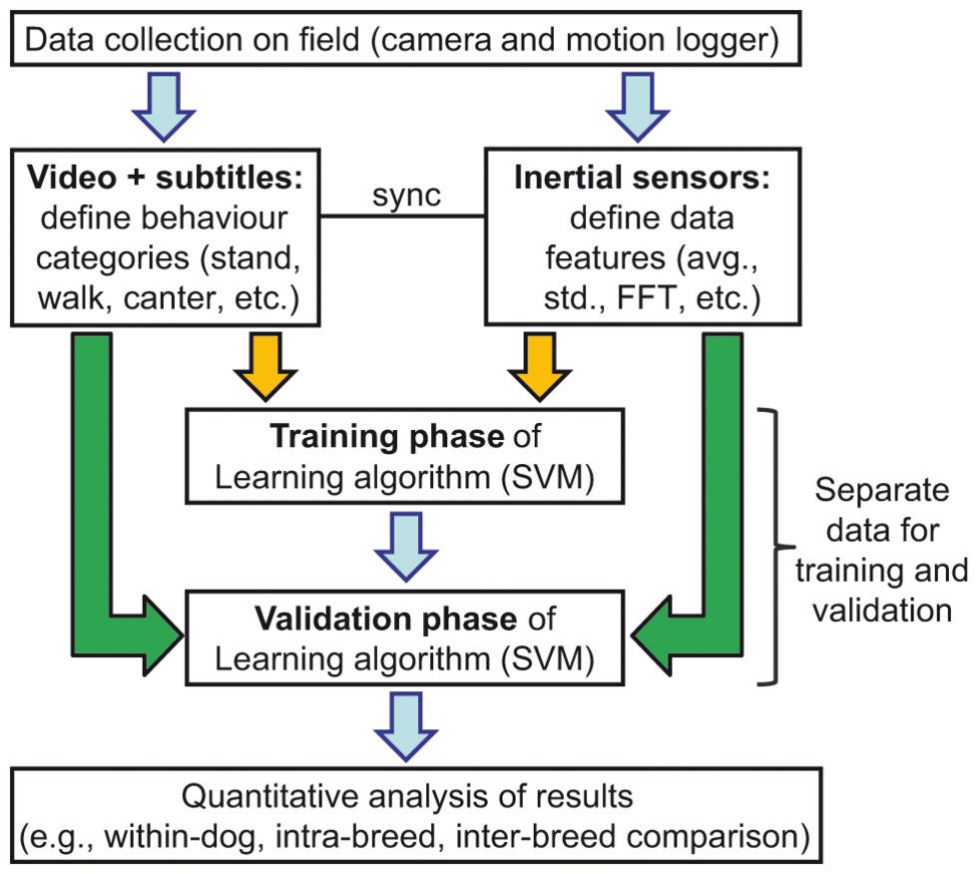
Schematic illustration of the procedure and data analysis (see text for details).

Figure 3 provides an overview of the workflow of data assessment and analysis. The time-stamped subtitle database (created by the main coder) served as the training data source for the offline supervised learning algorithm, while the raw video recordings were used as a visual feedback during the development and testing phases. To further reduce the possibility of false category assignments, in the final dataset we used a one second margin on both sides (beginning and end) of all category definitions (subtitles) that was simply neglected. SPSS for Windows Version 21 was used for statistical data analysis. 

### Supervised learning algorithm

We used the LIBSVM toolkit [44] to implement a support vector machine (SVM) based supervised learning algorithm for the dog motion pattern recognition. Each input node was a 126 dimensional feature vector, containing multivariate information about the last one second of the inertial data, including average, standard deviation, higher moments, extrema values, counts and Fast Fourier Transform components of all input vector components, vector lengths and vector dot products of the measured linear acceleration, angular velocity, and derived angular acceleration data and tangents of acceleration (see Table S2). All features were normalized to the [0, 1] range to achieve best recognition results.

In the SVM algorithm we used a standard Gaussian kernel. The two main parameters, soft margin (C) and kernel parameter (γ) were scanned in a wide range with five-fold cross-validation on several preliminary small datasets to find an optimal compromise between high performance and low generalization error. The final selected values for the two main parameters were C=16 and γ=0.001 as reasonably good general values for real validation purposes on all measurements (see Figure S1). Note that these parameters were fixed prior to the main measurements to prevent over-optimization and biased data handling.

### Training-validation comparisons

Further on by *training* we mean the process of feeding the supervised learning algorithm with input together with known output to tune model parameters, and the term *validation* will refer to the process of feeding new, unknown input to the system and comparing its prediction and reliability with known output. Note that we always separated validation data as a hold-out set instead of using cross-validation on the training and validation sets together. Although multifold cross-validation reduces error variance, using hold-out sets resembles more to a real scenario in a real-time application, and that is what we needed to optimize for. 

To test the prediction that behaviour identification is most successful when training and validation are both done on data collected from the same subject, and also, that the breed of the dog might have an influence on the results, we systematically grouped our data several various ways and carried out calculations accordingly. Nine different training-validation groups were created, differing in the nature of relation between the training and validating data. There were two major groups based on the number of measurements used for training; training on data of a single measurement (INS data with the corresponding subtitle tags, belonging to a single measurement of an individual) and training on data of multiple measurements (more INS data with the corresponding subtitle tags, belonging to different measurements of different individuals).

When training on a single measurement, the validating pair was either the other measurement of the same individual (*within-dog comparison*) or another measurement of a different individual (*between-dog comparison*). In the latter case, the training and validating data pairs belonged to either individuals from the same breed (*intra-breed comparison*), or they belonged to individuals from the two different breeds (*inter-breed comparison*).

**Figure 4:**
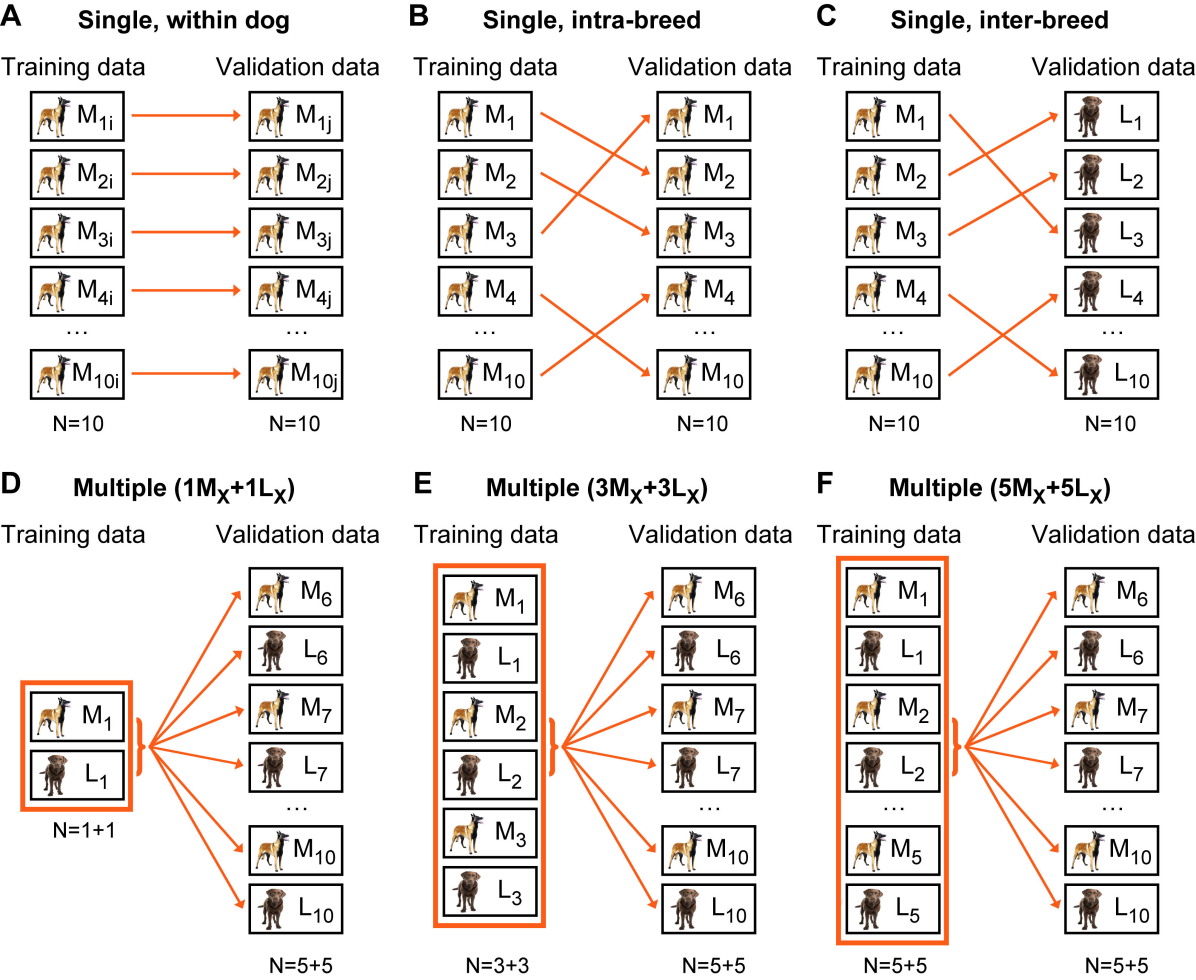
Illustration of the training-validation comparisons procedure. M stands for Malinois, L for Labrador breed, index numbers represent the individuals, i and j subscripts stand for the two subsequent measurements of the same individual. (A-C) The procedure of the three different cases of single dog training and validation, where one training process is always done on a single measurement and the outcome is validated on another independent measurement. Panels A-C only illustrate cases for Malinois as training and/or validation data, but the same procedures were done using the Labrador data as well. In the within dog comparison (panel A) the members of the training-validation pairs are two independent measurements of the same dog, one measurement used for training (M_Xi_ and L_Xi_), the other one of validation (M_Xj_ and L_Xj_) and there are ten pairs for both breeds. The intra-breed comparison (panel B) uses measurements of different individuals from the same breed as training-validation pairs (M_X_→M_Y_ and L_X_→L_Y_), while the inter-breed comparison (panel C) uses measurements of dogs from different breeds as training-validation pairs (M→L and L→M). (D-F) The three cases of multiple training comparisons with joint training data from several measurements. Panel D shows training with combined data of only two individuals (1M_X_+1L_X_), but larger training data sets containing 3+3 (E) or 5+5 (F) individuals are used as well. Throughout the three multiple cases the process of validation is always done on independent measurements of the same 5+5 (5M_Y_+5L_Y_) individuals, being different from the ones included in the training.

Training on multiple measurements was performed to test the efficacy of the present method as means of a more generalised, not strictly individual-specific automated behaviour identifying system. We extended the complexity of the training data by gradually increasing the number of the input measurements belonging to different individuals, and validated always on new data from other subjects that were not used in the training phase. Training and validation comparisons are illustrated on Figure 4.

We sorted all the single measurements according to the total number of input training nodes belonging to each of the seven discriminated behaviour categories. This *a priori* qualification was needed because even though the recorded dog activities followed the same protocol, the dogs were moving freely and their behaviour was not controlled strictly, resulting in individual differences in the ratio of the performed behaviours. The two distinct measurements of all our subjects were discriminated based on the minimum number of training nodes from all the seven behaviour categories. The one with higher absolute minimum was treated as the “more comprehensive” measurement and was used in all further analysis where possible. We ranked the more comprehensive measurements of all individuals as well based on this simple metric, and used the best twenty (ten Malinois and ten Labradors) for the selection of the training-validating pairs as described in more detail in the Supporting Information (Text S1).

### Feature importance

To assess and compare the information content of the 126 features extracted from the data, we created a summarized database of the best twenty (ten Malinois and ten Labradors) measurements. We calculated the F-score for all features, as a simple measure of feature importance [45]. Higher F-score corresponds to more information content. We performed five-fold cross-validation on the entire data, first with all features (126), then with features belonging to only the gyroscope (69) and only the accelerometer (45). For the availability of all original data and our analysis code see the Supporting Information (Text S2). 

### Intercoder agreement

Besides making a simple comparison of the raw data created by the main and secondary coders, we also checked how these data fit together within the training and validation processes. Ten calculations from the within-dog comparison (using data of five Labradors and five Malinois) were repeated three times, each in three different arrangements based on the alternation of subtitle labels of the main and secondary coders in the training and validation processes. The outcome of the above arrangements was compared to the outcome of the same calculations when both the training and validation phases were done with subtitle tags of the main coder.

## Results

### Training on single measurements

**Figure 5:**
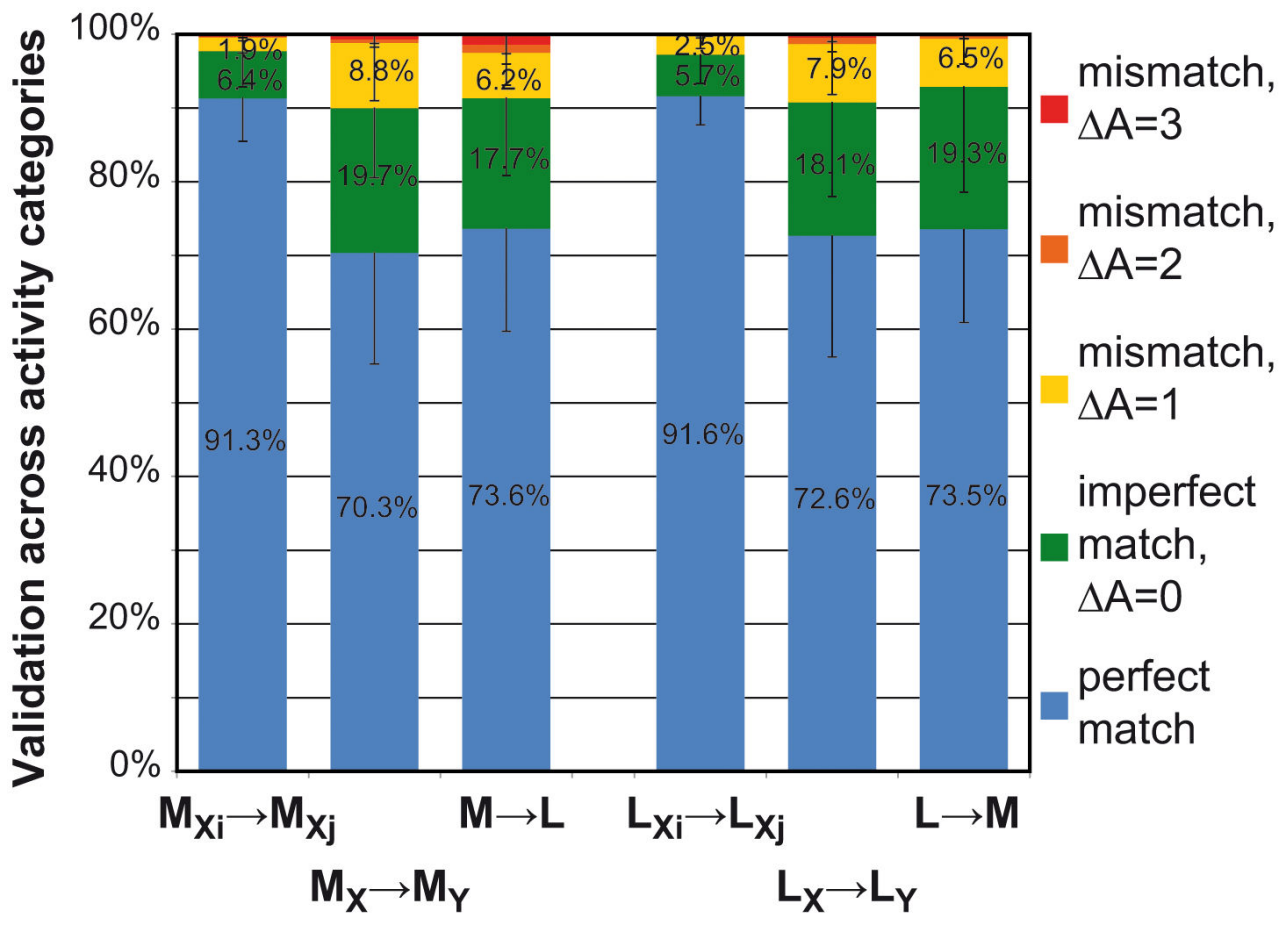
Validation accuracy (%) of within-dog, intra-breed and inter-breed comparisons. Data analysis was carried out by means of a supervised learning algorithm, using different combinations of independent sensor measurements for training and validation. The figure illustrates the results of six different training-validation comparisons (N=10 calculations in each comparison). The columns M_Xi_→M_Xj_ and L_Xi_→L_Xj_ indicate validation accuracies of within-dog, M_X_→M_Y_ and L_X_→L_Y_ intra-breed, M→L and L→M inter-breed comparisons. M stands for Malinois, L for Labradors, in the index X and Y represent different individuals, Xi and Xj stand for the two subsequent measurements of the same individual. Single measurements were used either for training (in prefix) or for validation (in suffix). Successful validation is indicated in blue (perfect match), false validations are further categorized according to the error in the activity level between the output and the input categories of behaviour (ΔA, where A_stand_=A_lay_=A_sit_=0, A_walk_=1, A_trot_=2, A_canter_=A_gallop_=3). Detection rate of all the seven categories (perfect match, in blue) is over 90% in within-dog comparisons, overall detection rate of the activity level (perfect and imperfect match, blue and green together) is over 90% for all comparisons.

Figure 5 illustrates the validation results from the single within-dog, intra-breed and inter-breed comparisons. Concerning the perfect matches (in blue), relationship between the training and the validating individual (i.e., same individual, same breed or different breed) had a significant effect on validation success (Mixed Model Analysis, F_2, 28.2_=24.200, p<0.001). Validation was most successful in the two within-dog comparisons, when both testing for Malinois and Labradors resulted in all the seven behavioural categories being identified correctly in 91.3% and 91.6% respectively. The ratios of correct identification in both the intra-breed (70.3% for M_X_-M_Y_ and 72.6% for L_X_-L_Y_) and inter-breed (73.6% for M_X_-L_X_ and 73.5% for L_X_-M_X_) comparisons were significantly lower (pairwise comparisons, p<0.001), while these two latter did not differ significantly from each other (pairwise comparisons, p=0.514). The breed of neither the training (Mixed Model Analysis, F_1, 15.463_=0.024, p=0.880) nor the validating (Mixed Model Analysis, F_1, 12.658_=0.052, p=0.823) individual had an influence the outcome of the tests. 

**Figure 6:**
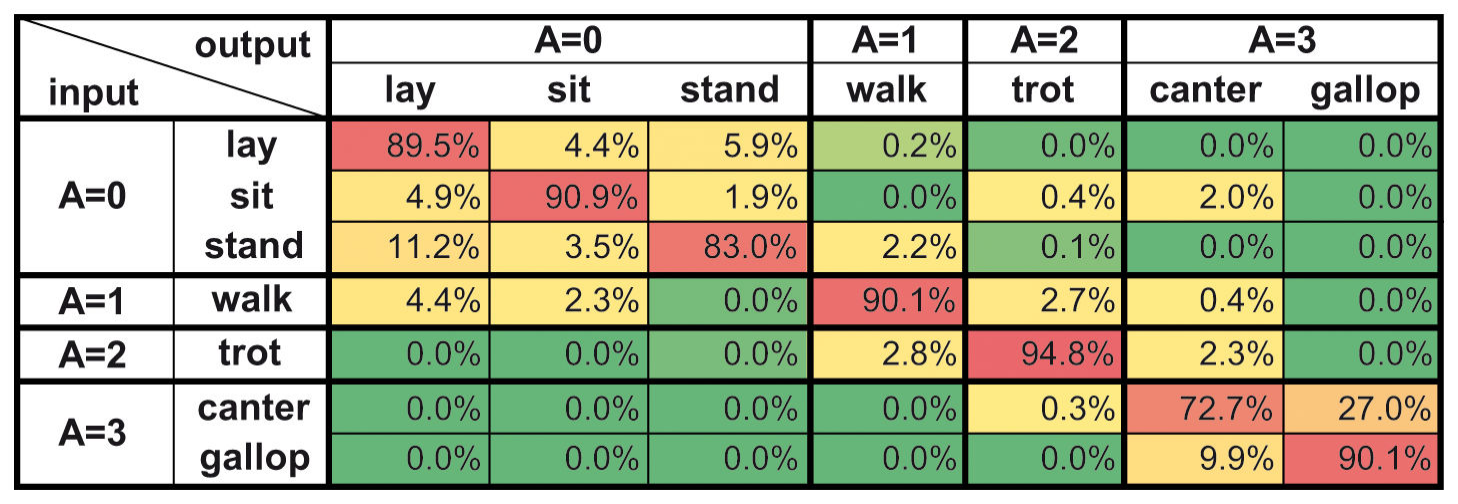
Recognition rate matrix of the within-dog comparison on Malinois (M_Xi_→M_Xj_). The figure is a detailed overview of the validation results of one single, within-dog comparison on Malinois (N=10 calculations) on the level of input and output behavioural categories. For more details about the given training and validating data see Figure 4 and the Supporting Information (Text S1). In the matrix, each row represents the training category (input) and each column represents the validation result (output) on the given training. Recognition rate values (%) are averaged for all calculations, thus they can be treated as probabilities of a given behavioural category recognized by the system as the same (diagonal) or another (off-diagonal) category (they add up to 100% in each row). Values are colour coded (green=low, yellow=mid-range, red=high recognition rate). *Ad* *hoc* activity levels (A) of all categories are shown on the first row and column. Diagonal elements represent perfect prediction (perfect match), near-diagonal elements are close to perfect (imperfect match) as they still belong to the same activity level. Mismatch of activity level is almost negligible.

We divided the non-perfect matches that occurred in validation into four subgroups. This was done according to an arbitrary measure based on the previously determined activity levels of the behavioural elements (see Table S1), as an extent of the failure (see Figure 5, imperfect match and mismatches). The interaction between the extent of the failure and the relationship between the training and validating individuals was significant (Mixed Model Analysis, F_3, 190.8_=5.72, p=0.001). In all the examined comparisons, the major part of the errors belonged to the imperfectly matched group, meaning that although perfect identification failed, the “assigned” behavioural category (the output) was on similar activity level as the “actual” input   element. Also, the ratio of failing by only one activity level (mismatch, ΔA=1 group) remained always higher than failing by two or three levels. For a more detailed overview of the validation results of one of the single, within-dog comparisons on the level of input and output behavioural categories, see Figure 6.

### Training on mixed, multiple measurements

**Figure 7:**
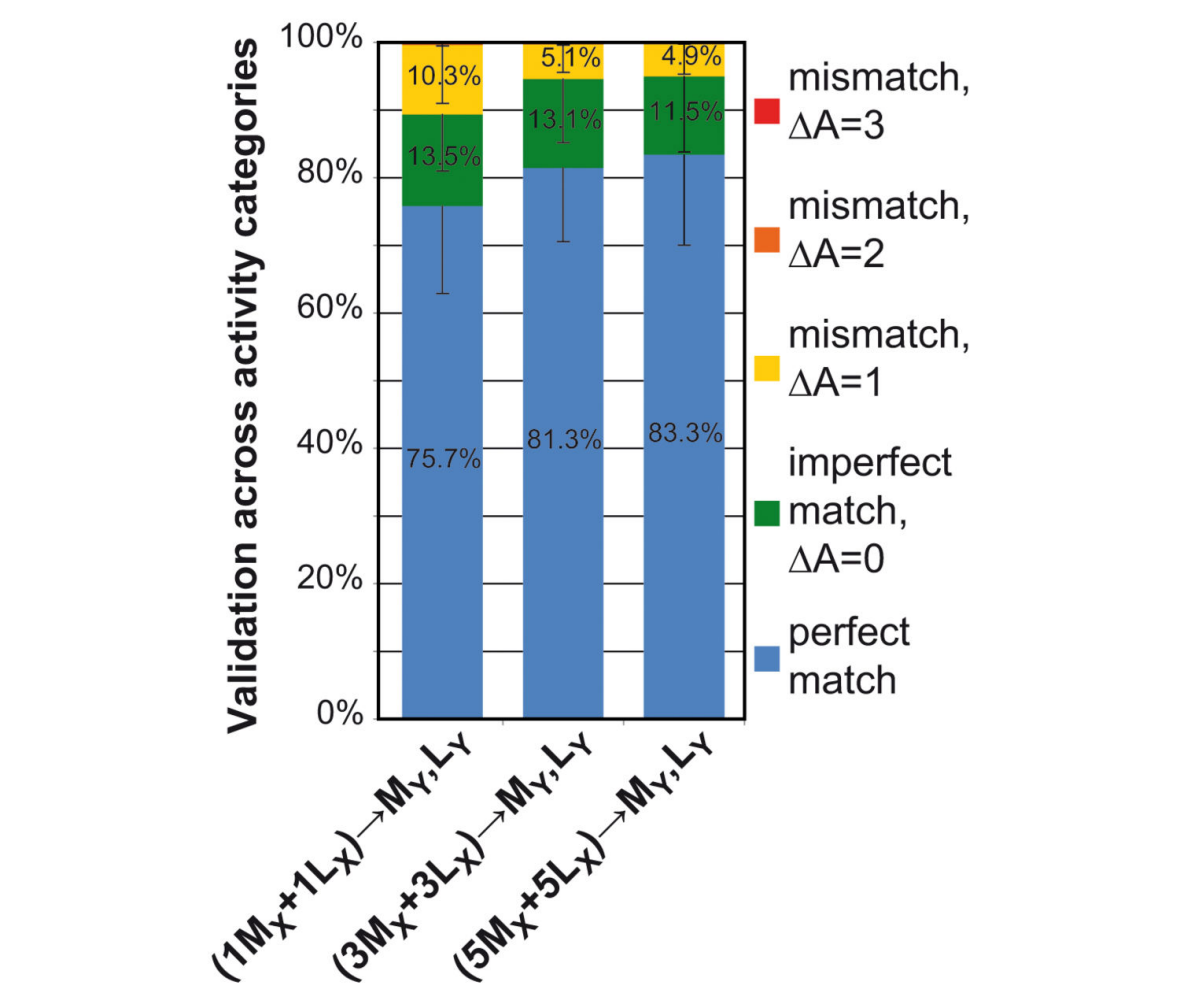
Validation accuracy (%) of the multiple training comparisons. Data analysis was carried out by means of a supervised learning algorithm, using different combinations of independent sensor measurements for training and validation. The complexity of the training data was also extended by gradually increasing the number of the input measurements. The figure illustrates the results of the three different multiple training comparisons (N=10 calculations in each comparison). The three columns correspond to the validation results of the three separate multiple training-validation groups constructed from 1M_X_+1L_X_, 3M_X_+3L_X_ and 5M_X_+5L_X_ as training measurements (in prefix). M stands for Malinois, L for Labradors, in the index X and Y represent different individuals. Validation of each group was carried out on the same ten individual measurements (M_Y_, L_Y,_ in suffix) not used in training. Successful validation is indicated in blue (perfect match). As the training set gets larger, the model gets more generalized and the recognition rate increases. False recognitions are further categorized according to activity level, for more details on that see legend of Figure 5.

The results of the three different multiple training groups are shown on Figure 7. Increasing the number of training measurements had an effect on validation success (Mixed Model Analysis, F_2, 18_=8.662, p=0.002). The mean ratio of correct identification of all the seven behavioural categories was 75.7% when training was done on two individuals of different breeds ((1M_X_+1L_X_)→M_Y_,L_Y_)). Compared to this, validation success increased significantly to 81.3% (pairwise comparisons, p=0.008) and further to 83.3% (pairwise comparisons, p=0.001) by changing the training input data to six ((3M_X_+3L_X_)→M_Y_,L_Y_), and ten individuals ((5M_X_+5L_X_)→M_Y_,L_Y_) respectively. These latter two groups, however, did not differ significantly from each other (pairwise comparisons, p=0.308). 

**Figure 8:**
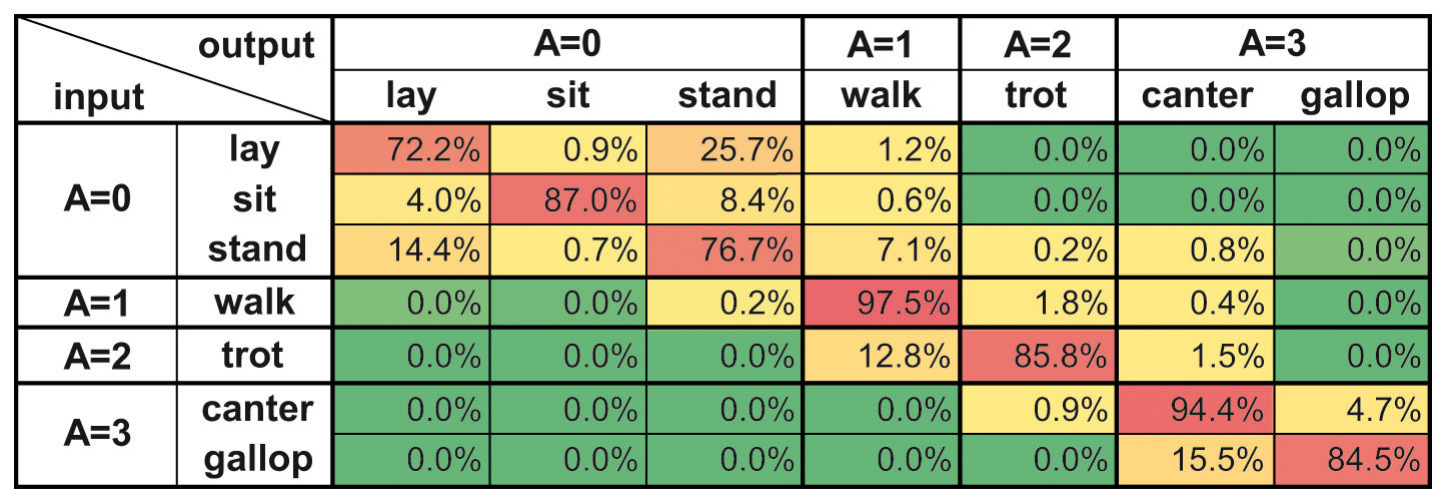
Recognition rate matrix of the (5M_X_+5L_X_)→M_Y_,L_Y_ multiple-training comparison. The figure is a detailed overview of the validation results of one multiple-training comparison (N=10 calculations) on the level of input and output behavioural categories. For more details about the given training and validating data see Figure 4 and the Supporting Information (Text S1). For further description about the structure of the matrix see legend of Figure 6.

Concerning validation failures, the major part of the mismatches belonged to the imperfect group in all the multiple training groups, as well. Again, the ratio of failing by only activity level remained higher than failing by two or three levels (see Figure 7). For a more detailed overview of the validation results of a multiple training group on the level of input and output behavioural categories, see Figure 8.

### Intercoder agreement

The reliability of the timestamped subtitle database was checked on twenty measurements that were also coded by a secondary coder. During the evaluation of the data we used our standard pre-processing steps as described in the method section (seven subtitle classes, one second margin etc.). Out of the total timestamped database of around 125,000 points for each coder, 20.8% and 18.0% were present in one coder’s database only. This high rate of difference in the input is mostly due to the effective pre-processing filter on videos with quick actions. From the remaining 102,813 data points with common timestamps from both coders, 96.8% were labelled the same way. More details about the misclassification are presented in the Supporting Information (Figure S2).

To test the non-common parts of the coders’ database as well and thus the robustness of the classification method, training and validation calculations were performed on the two coders’ data. No difference was found between the outcome of the within-dog training-validation comparisons carried out in four different arrangements, using either the subtitle tags of the main or the secondary coder for the training and/or the validation phases (General Linear Model, F_3, 27_=0.576, p=0.636)) (see Method and Table S3).

### Feature importance

**Figure 9:**
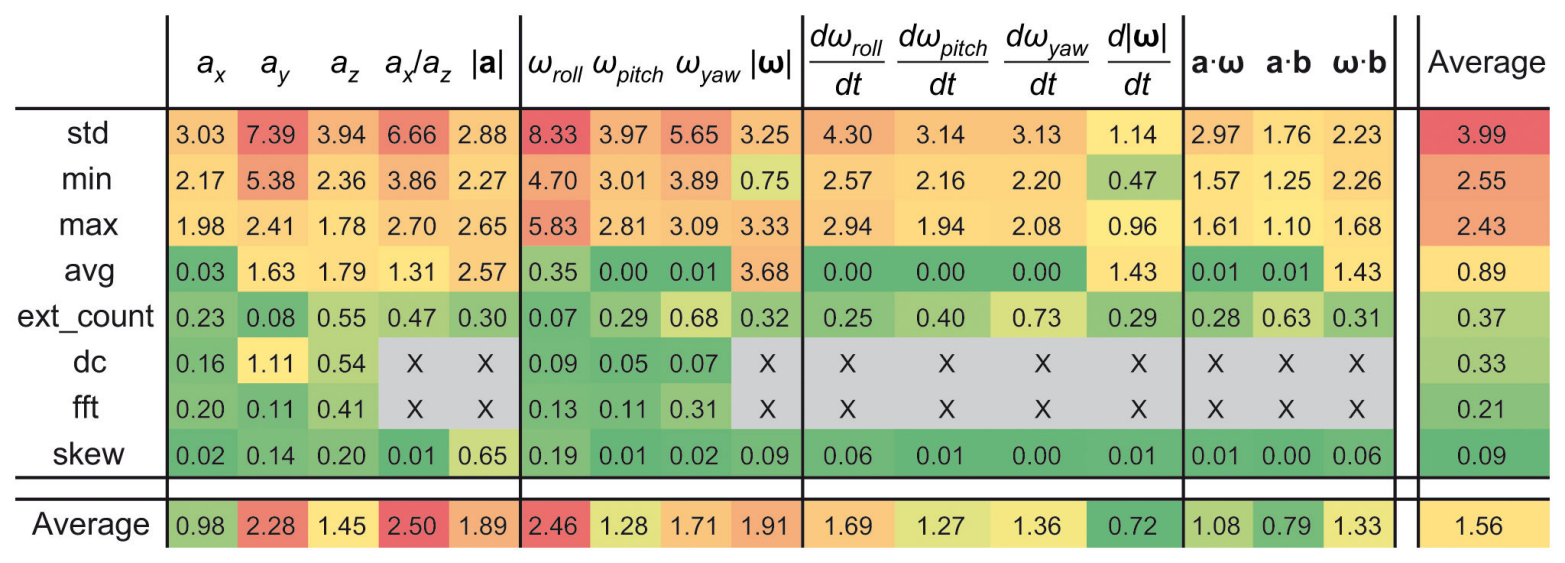
F-scores of input features. Features are categorized according to the base parameters and the calculated quantities (see Table S2 for details). Rows are ordered according to the average of the F-scores in them. Colour coding corresponds to F-scores (higher values are red, lower values are green). Higher F-score value represents higher feature importance (more information content).

F-scores of all data features calculated on the joint dataset are shown in Figure 9. According to the F-scores, both accelerometer and gyroscope data provided useful information. Standard deviation, minimum and maximum turned out to be the most relevant feature classes in general, corresponding mostly to the overall activity level of the behaviours. Features based on acceleration that describe the static attitude through the measurement of gravity also performed well, since they can differentiate between static postures. Figure S3 shows a visualization of the value distribution of two useful features. Cross-validation of the joint dataset resulted in 91.3% recognition accuracy, using only the accelerometer data reduced the accuracy to 88.1%, using only the gyroscope data reduced the accuracy to 75.3%.

## Discussion

The goal of the present study was to develop a bio-logging system capable of automatically differentiating between qualitatively different behavioural categories of the domestic dog. By means of a supervised learning algorithm, we managed to identify seven activities (stand, sit, lay, walk, trot, canter, gallop) with a considerably high accuracy above 90% when training and validation were done on the same individual. 

Supervised clustering algorithms aim at automatically unfolding patterns of behaviour based on categories defined by human observers. This first requires a manual calibration phase to assign the predictor variables for each category of behaviour. Therefore it is essential to have an accurate observer-based behaviour classification in order to reduce the possibility of implanting errors into the system at first place, caused by the limitations of human perception and attention. Thus, an automatic behaviour classifying system based on manual input cannot be expected to perform more accurately than a human observer. By comparing our validation results with the intercoder agreement, it can be concluded that the system we presented is capable of a performance compatible with human categorisation in the case of the investigated behaviour patterns. 

An alternative method would be to use unsupervised clustering algorithms first for automatically classifying behaviour into distinct units, and associate those with human categories only afterwards. The latter might be reasonably applied to species when direct observation of the animal is not always possible (e.g., flying, diving free-ranging animals) [13].

Adding a gyroscope besides the accelerometer seems to improve the effectiveness of our recognition system only slightly. However, it is likely that including more complex motion patterns (e.g. spinning around, weltering) in the analysis would benefit more from having the gyroscope data as well. Moreover, proceeding towards the development of an attitude and heading reference system (AHRS) [44] requires both sensors *inter alia*. A number of studies focused on data derived from accelerometers to determine the energy expenditure of animals due to movement [6,20,21,30]. This has useful implications from ecologic point of view (e.g., conservation issues). However, the goal of automated data collection from ethologic point of view is rather to give the most precise differentiation possible between qualitatively different behaviours. Applying 126 dimensional vectors as input node instead of the widely used measure of partial and/or overall DBA [19,25,29] allowed for a more detailed, finer scaled motion analysis. It might also be possible to optimize the system further by selecting the most appropriate features with the help of calculated F-scores or any other measure of feature importance. Optimization can reduce computation time and increase effectiveness. Nevertheless, SVM is usually successful in distinguishing between the input features on the basis of their usefulness and thus disregarding the least important ones. Our results (i.e., significant difference comparing within-dog and between-dog validation success) also highlight the existence of individual differences in fine-motion characteristics. Future applications might be able to take advantage of this and use similar methods for investigating individual-specificity of some motion patterns in more detail or recognise individuals based on the specific movement pattern. Depending on the sensitivity of the sensors applied some changes at the individual level of behaviour may be detected sooner on the basis of bio-logging data then by the human observer. Moreover, we believe that further developments of our present model – such as adding additional sensors (e.g., magnetometer, pressure sensor or microphone) to the system, or an additional device at a second anatomical point of the animal – would not only improve behaviour recognizing success but also allow the widening of the spectrum of behavioural categories to be identified. 

It seems also possible to improve the effectiveness of the present method as a more generalized behaviour identifying system by extending the complexity of the input data and increasing the robustness of the predictor features. This provides an effective way of compensating for probable error sources when fine-tuning of the whole system to one specific individual is not possible (e.g., free-ranging animals). The roughly similar body conformation of the two dog breeds used in this study might explain why no inter-breed difference was detected in the between-dog validations. Testing other dog breeds with different body conformations would reveal the feasibility of an expanded behaviour model that could be applied to this species in general. 

The detected behaviour misclassifications were not random, the majority of the mismatches occurred among pairs of categories belonging to the same ad hoc activity level. The most frequent failures were among the behavioural category pairs of stand-lay and canter-gallop (see Figures 6 and 8). During standing and laying postures the inertial sensor data show the same pattern, i.e., negligible angular velocity and static gravitational acceleration along the dorso-ventral axis, which can well explain the higher number of errors here. Canter and gallop are both characterized by very noisy, dynamic, random data that are hard to differentiate. This relative large uncertainty in the particular case may be explained by the difficulty in distinguishing between these two categories of fast movement even by human coders. The ratio of intercoder agreement was the lowest among canter-gallop pairs (see Figure S2), suggesting that there might have been be an original higher inaccuracy in the training data concerning these two behavioural categories, causing a consequential inaccuracy in validation.

Additional factors behind misclassifications can be the possibly occurring noises in the sensor signals [46]. These might be caused, for example, by some behavioural sub-categories(s) overlapping with the main ones. To reduce this source of error, our defined categories were all non-overlapping and only the objectively distinguishable ‘pure’ behaviours were coded, where possible. As for the postures (sit, lay, stand), however, it was not taken into account whether the dog was panting meanwhile or not. Panting – especially of high intensity – results in a constant movement of the dog’s body, which is well detectable by the sensors thus causing the above mentioned noise in the signal. One major difficulty with the accelerometer is that body acceleration and gravity are measured simultaneously, which becomes a problem when the logger on the animal is not perfectly fixed, i.e., its attitude relative to the dog’s back is not constant during or between measurements, or when the subject is not moving on plain ground [25]. To decouple gravity from body acceleration, reliable attitude estimation is needed, but it is not possible in 3D with only a gyroscope and an accelerometer. Therefore, in our next generation logger a magnetometer will also be included with which a full attitude and heading reference system (AHRS) [44] will be available. Experiments under different environmental (terrain) conditions are targeted as well. It should also be taken into account that temperature compensation of the inertial sensors could drastically reduce their intrinsic noise. Although we have a temperature sensor included in the current logger and temperature data is available for all past measurements, we did not carry out the offline temperature compensation of the input data yet, because the demonstration of our method proved successful without temperature compensation as well. 

It is important to note that the quick evaluation of the data by the supervised learning algorithm opens up additional possibilities for real-time implementations of the present model. Along with that, there are several areas that could benefit from a more detailed automatic analysis of dog behaviour. The veterinary field has already been experimenting with accelerometer-based activity detection [21,34-37]. Search and rescue teams or other professionals working with dogs could gain objective data on remote dog behaviour, moreover, the integration of a loudspeaker to the system raises the question of remote control possibilities of specially trained canines [7]. Besides these, open-field guard or shepherd dog behaviour could also be analysed without the need for direct human presence. 

The advantages of using bio-logging based behaviour analysing systems in laboratory animals along with, or instead of video cameras had already been discussed before [47,33]. The input nodes of the inertial data we used were not specifically selected for typifying dog movement characteristics, so it seems feasible to employ the whole system to other species, too. The device being small and light weight should not affect the natural way of motion even in animals of smaller body size and might be able to provide valuable information for the further enhancement of animal welfare in a wide range of species and circumstances. All in all, our method is a detailed and accurate behaviour identifying system compatible with the abilities of a human observer concerning the investigated behaviour categories. Furthermore, it opens up new directions in high-throughput automated ethology.

## Supporting Information

Figure S1 **Sample cross-validation results of a single measurement.** Cross-validation accuracy (indicated by the colour of the dots) highly depends on two main SVM kernel parameters, C and γ. Cross-validation of a single measurement can usually be tuned to achieve close to perfect recognition (red areas in the figure), but the corresponding parameter choice is typically over-optimized for that specific measurement. After testing a couple of initial measurements we choose C=16 and γ=0.001 as a good compromise for most of the tests and thus providing acceptable generalization capability.
(TIF)

Figure S2 **Intercoder classification distribution.** Each row represents the behaviour category created by the main coder, each column represents the corresponding category label of the secondary coder. The percentage values stand for all the commonly labelled data points of twenty measurements, thus they can be treated as probabilities of a given behaviour category of the main coder labelled as the same (diagonal) or another (off-diagonal) category by the secondary coder (they add up to 100% in each row). Values are colour coded (green=low, yellow=mid-range, red=high). Activity levels (A) of all categories are shown on the first row and column. Out of the ~125,000 data points 96.8% were classified in perfect agreement (diagonal elements in red). Moreover, differences overwhelmingly belong to the same activity level. Note that these results are similar to the training-validation results of the within-dog comparisons (see also Figure 6).
(TIF)

Figure S3 **Illustration of two features across all behaviour categories from all measurements.** Green dots represent the normalized values for the standard deviation calculated from the *x* axis of the gyroscope (std(*ω*_roll_); A) and for the minimum of the ratio between the *x* and *z* signals of the accelerometer (min(a*x*/a_*z*_); B) (within each category the vertical position of the dots is scattered with a random value for visibility). The probability density distributions are illustrated by the blue curves. std(*ω_roll_*) has the highest F-score, it provides the best differentiation between the categories, however it cannot distinguish between the static ones (A=0). min(a*x*/a_*z*_) has a best separation for those, as a proxy for attitude.
(TIF)

Table S1 **Detailed description of the recorded dog activities and definitions of the analysed behavioural categories with their corresponding ad-hoc activity levels (A).**
(DOCX)

Table S2 **Structure of the input nodes of the SVM algorithm.**
(DOCX)

Table S3 **Mean validation results (%) from within-dog comparisons (N=10 calculations in each) carried out in four different arrangements based on the alternation of subtitle labels of the main and secondary coder.** D_Xi_ and D_Xj_ stand for the two subsequent measurements of an individual (either Labrador or Malinois) used for either training (in prefix) or for validation (in suffix). MC indicates behaviour tags of the main coder while SC stands for behaviour tags of the secondary coder.
(DOCX)

Text S1 **Detailed description of the training-validation comparisons.**
(DOCX)

Text S2 **Data availability.**
(DOCX)

Video S1 **Illustration of the data collecting procedure.** The data collecting procedure consisted of 4 phases; (1) Preparation, (2) Sensor synchronisation, (3) Behaviour recording and (4) Sensor synchronisation. This video footage shows short sections from the Sensor synchronisation and Behaviour recording phases of one measurement with a Labrador recorded by a hand-held video camera. The video starts with the synchronization phase, showing first the time in UTC displayed by a laptop screen then the shaking of the logger on the dog’s back attached to its harness. During the following scenes the freely moving dog can be seen performing several different activities (sit, stand, gallop, canter, walk and trot) under the leadership of its handler. The video ends by parts from the second synchronisation phase. The added subtitles always indicate the actual phase, and the illustrated categories of the recorded behaviours are also marked during the behaviour recording phase.
(WMV)
