# Supplementary figures and images for: Identification of Behaviour in Freely Moving Dogs (Canis familiaris) Using Inertial Sensors
Source: PLoS One. 2013 Oct 18;8(10):e77814. doi: 10.1371/journal.pone.0077814 (PMC3820959; doi:10.1371/journal.pone.0077814)

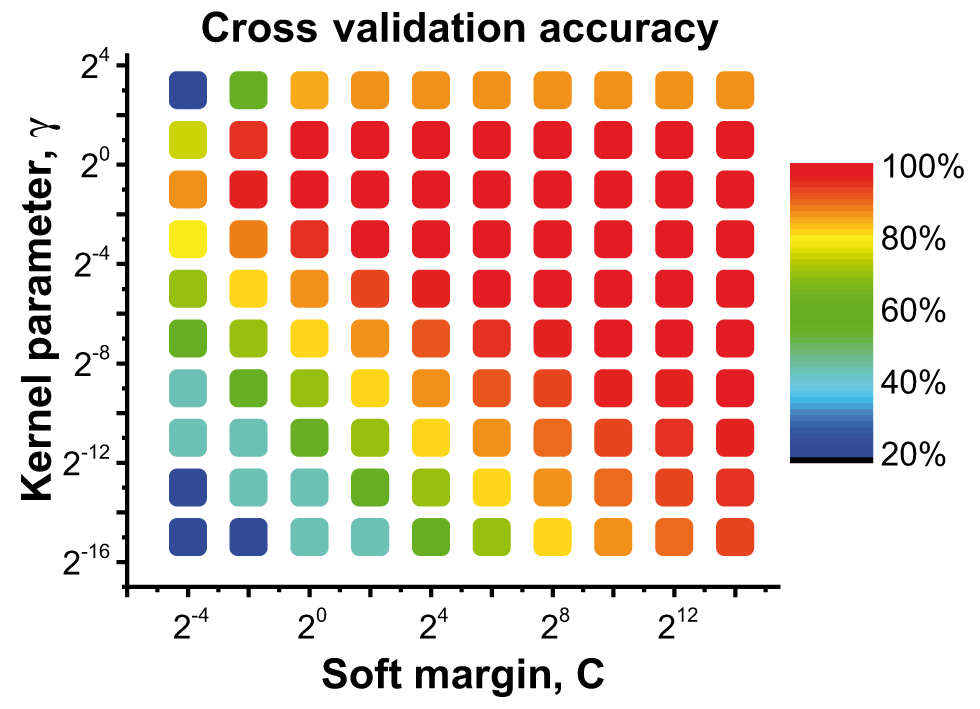

Supplement: Figure S1 — Sample cross-validation results of a single measurement. Cross-validation accuracy (indicated by the colour of the dots) highly depends on two main SVM kernel parameters, C and γ. Cross-validation of a single measurement can usually be tuned to achieve close to perfect recognition (red areas in the figure), but the corresponding parameter choice is typically over-optimized for that specific measurement. After testing a couple of initial measurements we choose C=16 and γ=0.001 as a good compromise for most of the tests and thus providing acceptable generalization capability. (TIF) [file pone.0077814.s001.tif]

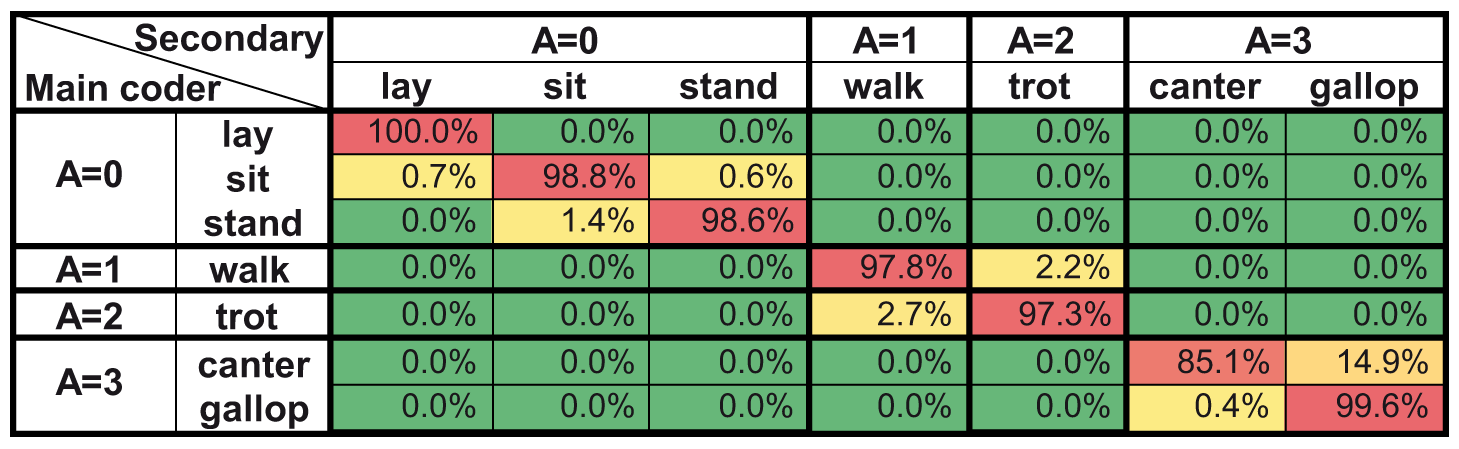

Supplement: Figure S2 — Intercoder classification distribution. Each row represents the behaviour category created by the main coder, each column represents the corresponding category label of the secondary coder. The percentage values stand for all the commonly labelled data points of twenty measurements, thus they can be treated as probabilities of a given behaviour category of the main coder labelled as the same (diagonal) or another (off-diagonal) category by the secondary coder (they add up to 100% in each row). Values are colour coded (green=low, yellow=mid-range, red=high). Activity levels (A) of all categories are shown on the first row and column. Out of the ~125,000 data points 96.8% were classified in perfect agreement (diagonal elements in red). Moreover, differences overwhelmingly belong to the same activity level. Note that these results are similar to the training-validation results of the within-dog comparisons (see also Figure 6). (TIF) [file pone.0077814.s002.tif]

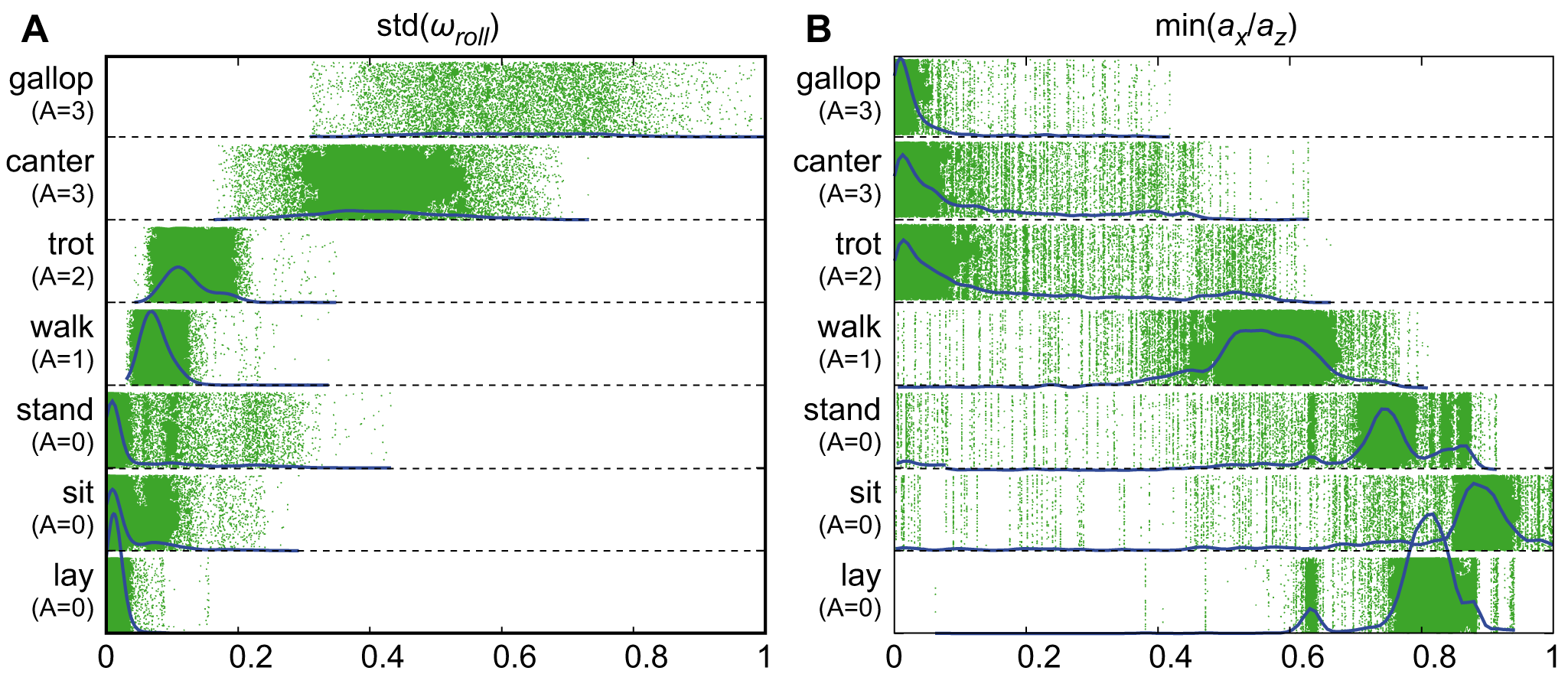

Supplement: Figure S3 — Illustration of two features across all behaviour categories from all measurements. Green dots represent the normalized values for the standard deviation calculated from the x axis of the gyroscope (std(ω roll); A) and for the minimum of the ratio between the x and z signals of the accelerometer (min(ax/az); B) (within each category the vertical position of the dots is scattered with a random value for visibility). The probability density distributions are illustrated by the blue curves. std(ωroll) has the highest F-score, it provides the best differentiation between the categories, however it cannot distinguish between the static ones (A=0). min(ax/az) has a best separation for those, as a proxy for attitude. (TIF) [file pone.0077814.s003.tif]
