# Supplementary material for: Identification of Behaviour in Freely Moving Dogs (Canis familiaris) Using Inertial Sensors
Source: PLoS One. 2013 Oct 18;8(10):e77814. doi: 10.1371/journal.pone.0077814 (PMC3820959; doi:10.1371/journal.pone.0077814)
Supplement: Table S1 — Detailed description of the recorded dog activities and definitions of the analysed behavioural categories with their corresponding ad-hoc activity levels (A). (DOCX) [file pone.0077814.s004.docx]

| **Name** | **Description of activity** | **Definition of the behavioural category** | **A** |
| --- | --- | --- | --- |
| **Stand** | The dog stands calmly without any displacement for approx. 10 seconds besides or in front of its handler. | The dog stays at one place with four straight legs touching the ground. Movements of the tail and the head are allowed for. | 0 |
| **Sit** | The dog sits calmly for approx. 10 seconds besides or in front of its handler. | The dog stays at one place with front legs in extended position, both hind legs bent and the metatarsal regions touch the ground. Movements of the tail and the head are allowed for. | 0 |
| **Lay** | The dog lays calmly for approx. 10 seconds besides or in front of its handler. | The dog stays at one place in sternal recumbency with all four legs bent and both elbows touching the ground. Movements of the tail and the head are allowed for. | 0 |
| **Walk** | The handler and the dog walk at a constant convenient speed side by side along a rectangular path (approx. 10x15 m) in both directions for approx. 1 minute. | The dog is using the slowest, symmetric four-beat gait, by which it supports its weight with its feet in the following sequence: front left limb, hind right limb, front right limb, hind right limb [1]. | 1 |
| **Trot** | The handler and the dog trot at a constant convenient speed side by side along a rectangular path (approx. 10x15 m) in both directions for approx. 1 minute. | The dog is performing a symmetric gait by which the diagonal pairs of limbs move almost simultaneously [2]. | 2 |
| **Run** | The dog runs at desired speed to fetch an object (e.g., ball) that is thrown away by the handler at maximum possible distance (25-35 m), and runs back to the handler with the ball in the mouth. This usually takes 15-20   seconds. |  | - |
| **Canter (slow gallop)** |  | The dog is performing an asymmetrical, three-beat gait, the pattern of which is a hind foot, the opposite hind foot and its front diagonal, followed by the other front foot and suspension, when present [2]. | 3 |
| **Gallop** |  | The dog is performing an asymmetrical, four-beat gait, when it supports its weight with its feet in the following sequence: right front leg, left front leg, right hind leg, left hind leg (it can happen that the two limbs left front and right hind hit the ground simultaneously). Just after taking off from the front left foot the dog achieves suspension [2]. | 3 |
| **Bark** | The dog is in either sitting or standing position, facing its handler and is barking continuously for approx. 10 seconds. |  | - |
| **Search** | The dog is instructed by the handler to stay in either sitting or laying position while the handler goes away in a distance of about 20-30 m to hide the dogs’ ball behind a tree, in a bush, etc. After the return of the handler the dog is let go to search for the hidden object and return it to the handler. The duration of this activity (usually between 0.5-2 minutes), as well as the meanwhile performed dog gaits are highly individual-dependent. |  | - |

**References**

[1] Coros S, Karpathy A, Jones B, Reveret L, Van De Panne M (2011) Locomotion skills for simulated quadrupeds. ACM Transactions on Graphics (TOG) 30: 59.

[2] Nunamaker D, Blauner P (1985) Normal and abnormal gait. Textbook of small animal orthopaedics International veterinary information service, USA.
