# Supplementary material for: Identification of Behaviour in Freely Moving Dogs (Canis familiaris) Using Inertial Sensors
Source: PLoS One. 2013 Oct 18;8(10):e77814. doi: 10.1371/journal.pone.0077814 (PMC3820959; doi:10.1371/journal.pone.0077814)
Supplement: Table S2 — Structure of the input nodes of the SVM algorithm. (DOCX) [file pone.0077814.s005.docx]

| **Base parameter source** | **Base parameter components** | **Comments** |
| --- | --- | --- |
| Linear acceleration (*a*) | *a*_x_, *a*_y_, *a*_z_, *a*_x_/*a*_z_, \|***a***\| | Accelerometer measures acceleration of gravity as well, thus fixed attitude of the sensor on the dog’s back is necessary for replicable measurements. *a*_x_/*a*_z_ is the tangent of the cranio-caudal direction relative to ground. |
| Angular velocity (*ω*) | *ω*_x_, *ω*_y_, *ω*_z_, \|***ω***\| | Fixed attitude is necessary for gyroscope as well, which measures angular velocity in body-fixed coordinate system. |
| Angular acceleration(*b*) | d*ω*_x_/d*t*, d*ω*_y_/d*t*, d*ω*_z_/d*t*, d\|***ω***\|/d*t* | Calculated as the numerical derivative of ω. |
| Dot products | ***a·b***, ***a·ω***, ***ω·b*** | Included without any special intention, but increases recognition rate slightly. |
| **Input node vector components** | **Definition (for any base parameter component *x*)** | **Comments** |
| 1st, 2nd and 3rd moments | *μ* = E[*x*] = ∑*x*/*n* *σ* = (E[(*x*–*μ*)^2^])^1/2^  *γ* = E[((*x*–*μ*)/*σ*)^3^] | i.e., average (mean), standard deviation, skewness |
| Extrema values | min(*x*), max(*x*), ext_count(*x*) | Ext_count is the total number of local minima and maxima. |
| Fast Fourier Transformation components | dc, low, mid and high part of FFT(*x*) | Low, mid and high parts are calculated on the lower half of the spectrum. The upper half is mostly empty or includes pure noise. Note that these components are similar to the generally used partial dynamic body acceleration, but FFT provides a more general context for accessing high frequency components. |
