# Supplementary material for: Identification of Behaviour in Freely Moving Dogs (Canis familiaris) Using Inertial Sensors
Source: PLoS One. 2013 Oct 18;8(10):e77814. doi: 10.1371/journal.pone.0077814 (PMC3820959; doi:10.1371/journal.pone.0077814)
Supplement: Table S3 — Mean validation results (%) from within-dog comparisons (N=10 calculations in each) carried out in four different arrangements based on the alternation of subtitle labels of the main and secondary coder. DXi and DXj stand for the two subsequent measurements of an individual (either Labrador or Malinois) used for either training (in prefix) or for validation (in suffix). MC indicates behaviour tags of the main coder while SC stands for behaviour tags of the secondary coder. (DOCX) [file pone.0077814.s006.docx]

|  | **D_Xi_(MC)**→**D_Xj_(MC)** (N=10) | **D_Xi_(SC)**→**D_Xj_(SC)** (N=10) | **D_Xi_(MC)**→**D_Xj_(SC)** (N=10) | **D_Xi_(SC)**→**D_Xj_(MC)** (N=10) |
| --- | --- | --- | --- | --- |
| **Mean (%)** | 92.2 | 90.9 | 90.8 | 92.1 |
| **SD** | 3.7 | 5.2 | 4.0 | 3.6 |
