# Supplementary material for: Identification of Behaviour in Freely Moving Dogs (Canis familiaris) Using Inertial Sensors
Source: PLoS One. 2013 Oct 18;8(10):e77814. doi: 10.1371/journal.pone.0077814 (PMC3820959; doi:10.1371/journal.pone.0077814)
Supplement: Text S1 — Detailed description of the training-validation comparisons. (DOCX) [file pone.0077814.s007.docx]

*Within-dog comparison*: Ten training-validation pairs were formed from subjects belonging to the same breed, and the whole process was done with both breeds, Malinois and Labradors. Since in this analysis both measurements of each individual had to be used, the more comprehensive (see the main text for details) was used for the training and the other one was used for the validation.

*Intra-breed comparison*: The more comprehensive measurement of one individual was used for training and the outcome was validated on the more comprehensive measurement of a different, randomly assigned individual belonging to the same breed. Ten such pairs were formed from subjects belonging to the same breed, resulting in altogether twenty cases, since the whole process was done with both breeds, Malinois and Labradors as well.

*Inter-breed comparison*: The more comprehensive measurement of one individual was used for training and the outcome was validated on the more comprehensive measurement of a different, randomly assigned individual belonging to the other breed. Ten such distinct training-validation pairs were formed, resulting in altogether twenty cases, since both breeds were used for training and validation, too.

*Multiple training comparisons*: From all the measurements of both dog breeds, the most comprehensive 5-3-1 measurements (with the highest minimum node-values) were chosen for training, which resulted in three multiple-training data sets (groups) consisting of altogether 10-6-2 individual measurements (5-3-1 from Malinois and 5-3-1 from Labradors, respectively). Validation for all (5M_X_+5L_X_), (3M_X_+3L_X_) and (1M_X_+1L_X_) training groups was done on the 5-5 more comprehensive measurements of both dog breeds that were not used for the training of the (5M_X_+5L_X_) case.
