# Supplementary material for: Identification of Behaviour in Freely Moving Dogs (Canis familiaris) Using Inertial Sensors
Source: PLoS One. 2013 Oct 18;8(10):e77814. doi: 10.1371/journal.pone.0077814 (PMC3820959; doi:10.1371/journal.pone.0077814)
Supplement: Text S2 — Data availability. (DOCX) [file pone.0077814.s008.docx]

All the data are available on request. Request should be sent to vasarhelyi@[hal.elte.hu](http://hal.elte.hu/).
